# Neonatal deaths in Cambodia: findings from a community-based mortality review

**DOI:** 10.1186/s13104-019-4265-5

**Published:** 2019-04-24

**Authors:** A. N. Bazzano, C. Var, D. Wilkosz, R. Duggal, R. A. Oberhelman

**Affiliations:** 1 Department of Global Community Health and Behavioral Sciences, Tulane University School of Public Health and Tropical Medicine, 1440 Canal St., New Orleans, LA 70112 USA; 2 National Institute of Public Health, #2 Kim Y Sung Blvd, Tuol Kork, P.O. Box 1300, Phnom Penh, Cambodia; 3 Reproductive Health Association of Cambodia (RHAC), #5, 150 St., Phnom Penh, Cambodia; 4 Tulane University School of Medicine, 1430 Tulane Ave, New Orleans, LA 70112 USA

**Keywords:** Infant, Newborn, Cambodia, Child mortality, Perinatal mortality, Health services

## Abstract

**Objectives:**

The aim of this study was to describe potential factors contributing to neonatal mortality in Takeo, Cambodia through assessment of verbal autopsies collected following newborn deaths in the community. The mortality review was nested within a trial of a behavioral intervention to improve newborn survival, and was conducted after the close of the trial, within the study setting. The World Health Organization standardized definition of neonatal mortality was employed, and two pediatricians independently reviewed data collected from each event to assign a cause of death.

**Results:**

Thirteen newborn deaths of infants born in health facilities participating in a community based, behavioral intervention were reported during February 2015–November 2016. Ten deaths (76.92%) were early neonatal deaths, two (15.38%) were late neonatal deaths, and one was a stillbirth. Five out of 13 deaths (38.46%) occurred within the first day of life. The largest single contributor to mortality was neonatal sepsis; six of 13 deaths (46.15%) were attributed to some form of sepsis. Twenty-three percent of deaths were attributed to asphyxia. The study highlights the continuing need to improve quality of care and infection prevention and control, and to fully address causes of sepsis, in order to effectively reduce mortality in the newborn period.

## Introduction

The first 28 days of life are critical for a child’s survival, when infants face the highest risk of mortality. In 2016, 46% of under-five deaths occurred during the neonatal period [1]. The vast majority of newborn deaths occur in low-income countries where access to health care is restricted. In Cambodia, neonatal mortality rates have dropped considerably in recent years, from 24 neonatal deaths per 1000 live births in 2009 to 18 per 1000 live births in 2014; however, regionally, the newborn mortality rate varies significantly, from a high of 36 neonatal deaths per 1000 live births in the Mondul Kiri and Ratank Kiri provinces, to a low of 12 per 1000 live births in Battambang and Pailin provinces [2]. The national rate is still nearly three times higher than the World Health Organization Western Pacific regional average of 6.5, and a child born in Cambodia is still significantly more likely to die than one born in a high-income country [1]. Formative research in the local setting found gaps in essential newborn care practices [3] and barriers to infection prevention and control [4]. The data from that formative research guided an intervention design for a clustered randomized trial, the Newborn Infection Control and Care Initiative (NICCI) trial [5]. Study objectives included improving infection control and referral systems in selected health centers, increasing knowledge and recognition of danger signs for sick newborns by mothers and families of newborn infants, and diagnosing the causes of sepsis among infants with possible sepsis at Takeo Province Hospital.

Following the end of the intervention, which took place over the course of 22 months, data on 13 neonatal deaths that had occurred within the study site had been collected. Researchers conducted verbal autopsy (VA) interviews using a standardized questionnaire with family members of newborns to ascertain cause of death, as local medical records on these were not available for review by study personnel. Per the World Health Organization (WHO), “Verbal autopsy is a method used to ascertain the cause of a death based on an interview with next of kin or other caregivers. This is done using a standardized questionnaire that elicits information on signs, symptoms, medical history and circumstances preceding death. The cause of death, or the sequence of causes that led to death, are assigned based on the data collected by a questionnaire and any other available information.” These interviews are used where the majority of deaths occur at home or where there is little chance that deaths will be recorded and causes of death determined [6].

The current study aimed to describe the factors potentially contributing to the 13 deaths in the study setting. Specifically, the study sought to analyze the verbal autopsies of perinatal deaths in both intervention and control groups, describe the timing and causes of these deaths, describe the basic demographics of newborns and mothers, and to understand factors surrounding these deaths.

## Main text

The study examining neonatal deaths was conducted through a retrospective review of 13 verbal autopsy reports. The standardized definition from the WHO for neonatal mortality was used as the inclusion criteria for this study. Local medical records on the deaths were not available for review. Two pediatricians reviewed verbal autopsy data and assigned a presumed cause of death, per WHO verbal autopsy guidelines [6]. Neonatal death was defined as death of a live-born child occurring within 28 days of life. Early neonatal death referred to death occurring within seven days of life while death occurring on or after seven days but before 28 days was referred to as late neonatal death [7]. Additionally, the WHO definition of intrapartum and very early neonatal mortality—occurring within the first 24 h of life and excluding newborns under 2500 g—was used to identify births that occurred due to intrapartum events [8].

### Setting

Takeo province is located in southern Cambodia, bordering Vietnam. The population, as of 2013, was 923,373[9]. There are eight national hospitals and in Takeo, there are 73 health centers, three primary referral hospitals, and one secondary referral hospital [10].

### Data collection and analysis

Verbal autopsy questionnaires were administered in Khmer language by trained members of the larger intervention study who had post secondary education in midwifery, during the process of monitoring newborn deaths in the study area. The questionnaire had both open and closed-ended questions and included portions that provided for respondent's verbatim account of the circumstances leading to the death of the child. Immediate caregivers (most often mothers) were the primary target respondents. The interviews were conducted on average of 2 months after death had occurred. Interviews lasted between 30 minutes and one hour and informed consent was obtained from all participants.

### Ethics approval and consent to participate

The study was approved by the National Ethics Committee on Health Research of the Cambodia Ministry of Health and by the Institutional Review Board of Tulane University. Informed consent to participate was obtained in writing from all particpants and no identifying information is presented in this manuscript requiring additional consent.

The parent study was registered with ClinicalTrials.gov, number NCT02271737. Descriptive data were extracted from 13 verbal autopsy forms reporting on deaths between February 2015 and November 2016. Analysis included summary statistics of causes and timing of deaths, as well as a qualitative description of circumstances surrounding the birth and death.

## Results

Thirteen newborn deaths occurred. Eight of those deaths (61.54%) occurred within the control group. Ten out of all 13 deaths (76.92%) were early neonatal deaths, two (15.38%) were late neonatal deaths, and one was a stillbirth. The largest single contributor to neonatal death in the sample, per cause of death assigned by consulting pediatricians, was neonatal sepsis. Six out of 13 deaths (46.15%) were attributed to some form of sepsis. Twenty-three percent of the deaths were attributed to asphyxia. Other causes of death included stillbirth and prematurity.

The majority of the newborns (76.92%) were male, and their mothers’ ages ranged from 19 to 35, whom were married between the ages of 17 and 27. The mothers had an average of 6.7 years of schooling and their average household size was 6.4 people per home.

The number of mothers’ antenatal care visits ranged from two to nine, with an average of five. At those visits, only six women were informed of any danger signs to be aware of during pregnancy or where to go if experiencing any of those signs.

All 13 newborns were born in a health facility, and five (38.46%) died at the same facility in which they were born. Of the eight babies who went home after birth, four (50%) were referred to a hospital upon discharge of the facility where they were born; all four died in the hospital after referral. Of those mother/baby pairs who went home after birth, none of them reported being visited by a community health worker at home.

Twelve out of 13 (92.31%) deliveries were vaginal, with the exception being the stillbirth, where cesarean section was used to deliver the baby. Of the 12 vaginal deliveries, three (25%) were with forceps. Four of the 13 babies (23.08%) were ever breastfed, and seven of 13 (53.85%) were fed either a pre-mixed formula or powdered formula mixed with a liquid. Three out of 13 mothers (23.08%) received counselling upon discharge of the birth facility that did *not* include referral to a hospital; two of those three were in the intervention group.

Ten out of the 13 recorded deaths (76.92%) were within the first week of life, five of which (38.46%) occurred between zero and one day. Four out of the 10 early neonatal deaths (40%) were within the intervention group. The largest contributors to early neonatal death in our sample were neonatal sepsis and asphyxia. Three of the 10 early neonatal deaths (30%) were due to neonatal sepsis and three more of the 10 (30%) were due to asphyxia. Other causes of early neonatal death recorded were other/unspecified, pneumonia, and prematurity. Two of the 13 recorded deaths (15.38%) occurred between the 7th and 28th day of life—one from the control group and one from the intervention group. Neonatal sepsis was the cause of death in both cases.

We report on the causes of deaths of 13 neonates following verbal autopsy interviews with family members of the deceased. Five of the deaths reported (38.46%) were linked to intrapartum events, including four of the five deaths that occurred within the intervention group. Severe infection was the most common cause of death, followed by asphyxia.

Verbal autopsy analyses conducted in other countries indicate that sepsis and asphyxia are often among the top three causes of neonatal deaths. When conducting verbal autopsies in Morang, Nepal, Khanal et al. found that infection (41%), birth asphyxia (37%), and prematurity or low birth weight (18.4%) were the most common causes of neonatal death [11]. An analysis of verbal autopsies in Nigeria found that sepsis (31.5%), birth injury/asphyxia (22.3%), and pneumonia (19.9%) were the largest contributors to death [12]. A verbal and social autopsy (VASA) investigation conducted by the World Health Organization and UNICEF to estimate the causes of neonatal and child deaths in several high priority countries found that severe neonatal infection and asphyxia were the leading causes of neonatal death in Niger in 2010 [13].

One study identified an important linkage to mother’s age—more than half of recorded stillbirths in that study were to women under 21 years old, and babies born to mothers under 25 years old were less likely to survive compared to older mothers [14]. Research in Bangladesh and Malawi found delays in care-seeking to be an underlying cause of neonatal deaths [15, 16]. In the current study, only one verbal autopsy reported pre-term delivery and there was limited information on care-seeking, likely related to the small sample size.

Prompt initation and exclusive breastfeeding, which are known to protect against neonatal illness including sepsis [17, 18] were not widely practiced among study particpants, with only five families reporting breastfeeding of baby before death.

Additionally, the VA interviews highlight several important gaps regarding referral and postnatal care. First, no respondent who left the facility where she gave birth reported being visited by a community health worker at home, pointing to a lack of care coordination between health center staff and village health volunteers. Second, while seven mothers received some form of counselling upon discharge, four of those women were referred to a hospital, and of the three mothers who received counselling other than a hospital referral, only two received information regarding danger signs of newborn illness.

## Limitations

This study’s limitations include the small number of cases identified, the inability to review local medical records related to the newborn deaths, and the descriptive, retrospective nature of this study. Additionally, four of the respondents interviewed were not the mothers of the deceased (but rather were close family members who confirmed being present for pregnancy, delivery, and during the child’s illness and death) which may have impacted recall. Finally, some VA records contained very limited narrative detail, indicating that participants in the interviews were not able to provide comprehensive responses. Limited information such as this is common in community-based studies, and is to be expected given the sensitive nature and length of verbal autopsy interviews, but nonetheless restricts comprehensive analysis.

Improving quality of neonatal care is critical to reaching the Sustainable Development Goals [19]. This study sought to explore the continuing challenges related to perinatal survival in Takeo province, within the context of an intervention that aimed to improve care practices, coordination, and the recognition of danger signs of newborn illness.

All of the parents interviewed in our sample sought care for their newborns at local health centers, and the majority of newborn deaths were early neonatal deaths. Improving quality of care and health center protocols for managing birth complications at small health facilities must be a priority. For example, being able to identify and assist a newborn suffering birth asphyxia can reduce mortality–asphyxia accounted for 23% of the deaths in our sample [20]. In addition, nearly half of deaths in this study were due to some form of neonatal sepsis, indicating infection prevention control at the level of the health centers may have been inadequate. Sub-optimal infection prevention and control (IPC) practices have been identified in other settings as important contributors to mortality and morbidity [21]. In addition, more than half of the babies in our sample were never breastfed, and upon discharge only three women received any type of counselling, which includes advice on practices such as breastfeeding.

A systematic approach to understanding, identifying, and managing barriers to improving quality of care for neonatal illness is critical to ensuring that Cambodia’s trend of reducing neonatal mortality continues.

## Abbreviations

NICCI: Newborn Infection Control and Care Initiative trial
VASA: verbal and social autopsy
VA: verbal autopsy
WHO: World Health Organization
